# Rational Design of a Live Attenuated Dengue Vaccine: 2′-*O*-Methyltransferase Mutants Are Highly Attenuated and Immunogenic in Mice and Macaques

**DOI:** 10.1371/journal.ppat.1003521

**Published:** 2013-08-01

**Authors:** Roland Züst, Hongping Dong, Xiao-Feng Li, David C. Chang, Bo Zhang, Thavamalar Balakrishnan, Ying-Xiu Toh, Tao Jiang, Shi-Hua Li, Yong-Qiang Deng, Brett R. Ellis, Esther M. Ellis, Michael Poidinger, Francesca Zolezzi, Cheng-Feng Qin, Pei-Yong Shi, Katja Fink

**Affiliations:** 1 Singapore Immunology Network, Agency for Science, Technology and Research, Singapore; 2 Novartis Institute for Tropical Diseases, Singapore; 3 State Key Laboratory of Pathogen and Biosecurity, Beijing Institute of Microbiology and Epidemiology, Beijing, China; 4 State Key Laboratory of Virology, Wuhan Institute of Virology, Chinese Academy of Sciences, Wuhan, China; 5 Duke Graduate Medical School, Singapore; 6 Wadsworth Center, New York State Department of Health, Albany, New York, United States of America; University of Washington, United States of America

## Abstract

Dengue virus is transmitted by *Aedes* mosquitoes and infects at least 100 million people every year. Progressive urbanization in Asia and South-Central America and the geographic expansion of *Aedes* mosquito habitats have accelerated the global spread of dengue, resulting in a continuously increasing number of cases. A cost-effective, safe vaccine conferring protection with ideally a single injection could stop dengue transmission. Current vaccine candidates require several booster injections or do not provide protection against all four serotypes. Here we demonstrate that dengue virus mutants lacking 2′-*O*-methyltransferase activity are highly sensitive to type I IFN inhibition. The mutant viruses are attenuated in mice and rhesus monkeys and elicit a strong adaptive immune response. Monkeys immunized with a single dose of 2′-*O*-methyltransferase mutant virus showed 100% sero-conversion even when a dose as low as 1,000 plaque forming units was administrated. Animals were fully protected against a homologous challenge. Furthermore, mosquitoes feeding on blood containing the mutant virus were not infected, whereas those feeding on blood containing wild-type virus were infected and thus able to transmit it. These results show the potential of 2′-*O*-methyltransferase mutant virus as a safe, rationally designed dengue vaccine that restrains itself due to the increased susceptibility to the host's innate immune response.

## Author Summary

The four serotypes of dengue virus cause severe outbreaks globally in tropical countries with thousands of patients requiring hospitalization. The health care and indirect economic cost of dengue in endemic countries is huge. Despite this, no clinically approved vaccine or antiviral treatment is currently available. Dengue transmission could be stopped with a vaccine that provides full protection to all serotypes. Dengue afflicts many developing countries and a vaccine should therefore be cost-effective and should provide protection with ideally a single injection. Here we present a novel dengue vaccine approach that harbours mutation(s) in the 2′-*O*-methyltransferase (MTase), a viral enzyme that methylates viral RNA as a strategy to escape the host immune response. Non-methylated RNA is recognized as “foreign” and triggers an interferon response in the cell. The MTase mutant virus is immediately recognized by the host's immune response and hardly has a chance to spread in the organism while an immune response is efficiently triggered by the initially infected cells. Mice and monkeys infected with the mutant virus developed an immune response that fully protected them from a challenge with wild-type virus. Furthermore, we show that MTase mutant dengue virus cannot infect *Aedes* mosquitoes. Collectively, the results suggest 2′-*O*-MTase mutant dengue virus as a safe, highly immunogenic vaccine approach.

## Introduction

Dengue virus (DENV) is a member of the *Flaviviridae* family. DENV infection causes dengue fever (DF) and the more severe forms of the disease, dengue hemorrhagic fever (DHF) and dengue shock syndrome (DSS). DENV has four serotypes (DENV-1 to -4), each of which is capable of causing severe disease. The frequency, severity, and geographical spread of cases have increased over the past decades [1], [2]. Every year, one hundred million new cases of DF and 250,000 DHF/DSS are estimated by the WHO. At present, despite intensive global research efforts, no vaccine or antiviral treatment for dengue infection is available. Vaccine development is complex due to multiple factors. (i) An effective vaccine must consist of a tetravalent formulation protecting against each of the four serotypes because more than one serotype typically circulates in a region. (ii) A sub-protective vaccine potentially increases the risk of vaccinees to develop the more severe forms of dengue during repeated infection because of a known association of pre-existing immunity with severity [3], [4]. (iii) Since most infections occur in developing countries, an ideal vaccine should be affordable and fully protective [5]. Taken together, a vaccine inducing a robust level of immunity ideally with only one inoculation is required.

Live-attenuated vaccines are replication-competent viruses, which can induce an immune response and an immune memory that are comparable to those induced by the wild-type virus. Live-attenuated viruses do not cause disease because of the low level of replication and hence low levels of inflammation. Prominent examples of successful live-attenuated vaccines providing long-term immunity are those against vaccinia virus, poliovirus (Sabin), and two members of the *Flaviviridae* family, yellow fever virus (YF-17D) and Japanese encephalitis virus (JEV SA14-14-2) [6]. Live-attenuated DENV vaccines have been shown to induce protective neutralizing antibody titers in mice, monkeys, and humans [7]–[9]. In addition, evidence that a balanced T cell response contributes to protection is accumulating, emphasizing the importance of T cell epitopes in a vaccine [8].

Flaviviruses are positive-sense, single-stranded RNA viruses. The flavivirus genome encodes for 3 structural (C, prM, and E) and 7 non-structural proteins (NS1, NS2A, NS2B, NS3, NS4A, NS4B, and NS5). NS5 is a multifunctional protein, consisting of the RNA-dependent RNA polymerase [10] and methyltransferase (MTase) activities responsible for 5′ RNA cap formation [11], [12] as well as internal RNA methylation [13]. While N-7-methylation is essential for RNA translation and stability, the function of 2′-*O*-methylation has remained elusive until recently. We and others demonstrated that while 2′-*O*-MTase is not essential for viral replication *in vitro*, viruses bearing mutations in the highly conserved MTase catalytic K-D-K-E tetrad are severely attenuated in the host due to the inability of the virus to shield viral RNA from recognition by host innate immune factors [14], [15]. DENV RNA binds to RIG-I and MDA5 [16], [17], which activates interferon (IFN)-β production via a cascade involving IFN-β promoter stimulator 1 (IPS-1) [17]. IFNs in turn activate IFN stimulated genes (ISGs), which induce antiviral responses in infected and neighbouring cells. IFN-induced proteins with tetratricopeptide repeats (IFITs) are critical for the inhibition of viral infections, although their functions are only partially understood [14], [18]. The human IFIT gene family comprises four members: IFIT1, IFIT2, IFIT3 ( = IFIT4), and IFIT5; whereas mice only express IFIT1, 2 and 3 [19]. Interestingly, IFIT homologs are conserved from amphibians to mammals, suggesting that they play a central role in the innate immune response [19]. IFIT1 and 2 bind to eukaryotic Initiation Factor 3 (eIF3) and inhibit translation [20], whereas IFIT3 amplifies the antiviral signal by connecting IPS-1 and TBK1, resulting in more IFN production [21]. The role of human IFIT5 is less well understood.

Here we demonstrate that DENV strains bearing a mutation in the catalytic site of the 2′-*O*-MTase replicated to high titres in cell culture whereas these mutant viruses were highly attenuated in mice and rhesus monkeys. The mutation was stable over several passages and reversion to wild-type (WT) was not observed. For further safety improvement, a second mutation in the 2′-*O*-MTase catalytic tetrad was introduced without affecting the viability of the virus *in vitro*. A single dose administration to rhesus macaques (RM) conferred protection to homologous DENV challenge. Mice immunized with a single dose of a divalent (DENV-1/2) formulation of the mutant viruses and mice immunized with the monovalent formulation showed comparable antibody responses, demonstrating that there was no interference between two serotypes of the DENV MTase mutants. Moreover, no enhanced infection and increased TNF-α levels were observed in immunized mice upon challenge with heterologous virus. Overexpression of IFITs in HEK-DC-SIGN cells suggested a role for IFIT1 in the attenuation of MTase mutant in human cells. Taken together, these results demonstrate the potential of 2′-*O*-MTase mutants as a DENV vaccine. To our knowledge, this is the first live-attenuated rational vaccine approach, tailored to optimally activate the innate and adaptive immune response while being severely attenuated due to its susceptibility to the IFN response.

## Results

### N7- and 2′-*O*-methylation activities of WT and mutant DENV-1 and -2

**Figure 1:**
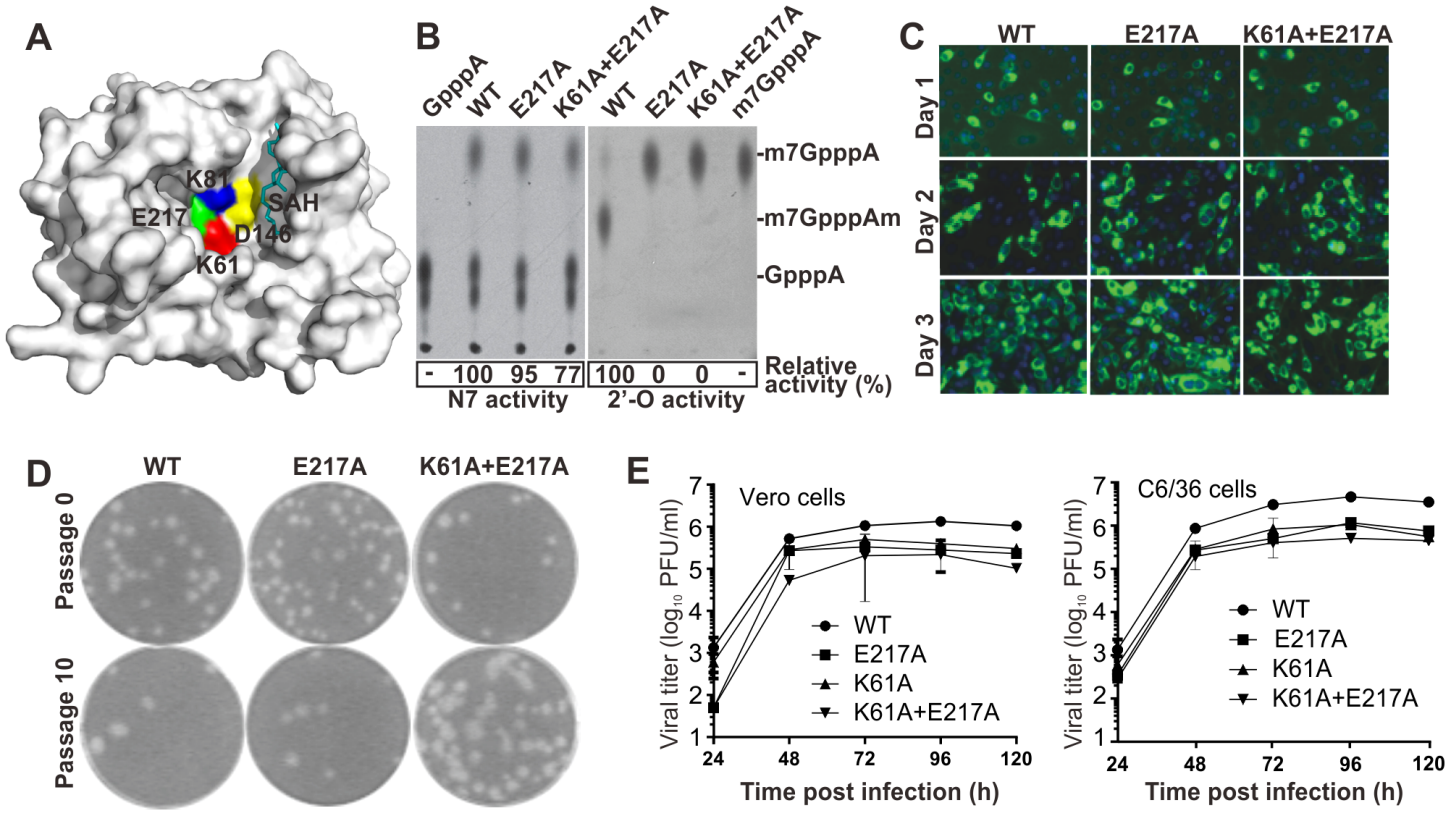
Characterization of DENV-2 MTase. (A) Surface representation of DENV-2 MTase structure showing active site residues K61 (red), K81 (blue), D146 (yellow), and E217 (green). SAH (*S*-adenosyl-L-homocysteine), a by-product of methylation reaction, is shown in stick (cyan). The image was prepared using DENV-2 MTase (PDB code: IL9K) [12] and PyMOL. (B) Effects of E217 and K61+E217A mutations on N7- and 2′-*O*-MTase activities. Recombinant MTases were assayed for GpppA-RNA→m7GpppA-RNA and m7GpppA-RNA→m7GpppAm-RNA conversions to indicate N7- and 2′-*O*-methylation activities, respectively. The reactions were analyzed on thin-layer chromatography (TLC) plates [11]. Relative methylation activities were indicated below the TLC images with WT activity set as 100%. (C) Immunofluorescence analysis (IFA). BHK-21 cells were electroporated with equal amounts of WT and mutant genome-length RNAs of DENV-2. At indicated days post transfection, the cells were subjected to IFA using mouse antibody 4G2 against DENV E protein and anti-mouse IgG conjugated with FITC as primary and secondary antibodies, respectively [23]. (D) Plaque morphology. WT and mutant viruses recovered from genome-length RNA-transfected cells (passage 0), as well as the viruses after culturing on Vero cells for 10 rounds (passage 10) were analyzed by plaque assays [23]. (E) Growth kinetics. Vero and C3/36 cells were infected with WT and mutant DENV-2 at a multiplicity of infection (MOI) of 0.1. Viral titers were measured at indicated time points using plaque assays. Average results of three experiments are presented.

Flaviviruses replicate in the cytoplasm. The cytoplasm-replicating viruses have evolved N7- and 2′-O-methyltransferases (MTase) to methylate their viral mRNA 5′ cap structures [22]. We have previously shown for West Nile virus (WNV) and DENV-1 that mutation of the Asp of the tetrad K-D-K-E completely abolished both N7- and 2′-*O*-MTase activities and was lethal for viral replication; mutations of the other three residues of the tetrad abolished 2′-*O*-methylation (with a decrease in N7-methylation), and led to attenuated viruses [14], [23]. Since there are four serotypes of DENV, we introduced the same MTase mutations into DENV-2 to examine whether the same approach was feasible with more than one serotype. A WT recombinant MTase, representing the N-terminal 296 amino acids of the DENV-2 NS5 (strain TSV01), was cloned and expressed. Two mutant MTases containing Ala-substitutions at the K-D-K-E tetrad (Fig. 1A) were prepared: one with a single E217A mutation and another with double K61A+E217A mutations. The mutant enzymes retained 95% and 77% of the WT N7-methylation activity, respectively; neither mutant exhibited any 2′-*O*-methylation activity (Fig. 1B). BHK-21 cells transfected with equal amounts of WT and mutant (E217A and K61A+E217A) genome-length RNAs of DENV-2 generated equivalent numbers of viral E protein-expressing cells (Fig. 1C). Both WT and mutant RNAs produced infectious viruses (passage 0) with similar plaque morphologies (Fig. 1D). The replication of mutant viruses was attenuated in mammalian Vero and mosquito C3/36 cells (Fig. 1E). Continuous culturing of the mutant viruses on Vero cells or HEK-293 cells expressing DC-SIGN (HEK-DC-SIGN) for ten rounds (3–4 days per round) did not change their plaque morphologies (Fig. 1D and data not shown). The expression of DC-SIGN facilitates DENV infection [24]. Sequencing of the passage 0 and 10 viruses from both Vero and HEK-DC-SIGN cells showed that the engineered mutations were retained (Supplementary Fig. S1a and S1b). Similar results were obtained for DENV-1 containing the E216A (E216 in DENV-1 MTase is equivalent to E217 in DENV-2 MTase) or K61A+E216A mutation in MTase (Supplementary Fig. S2). Collectively, the results demonstrate that the 2′-*O*-MTase mutant DENV-1 and -2 are slightly attenuated, but stable in cell culture.

### DENV 2′-*O*-MTase mutants are attenuated in mice and induce a protective immune response

**Figure 2:**
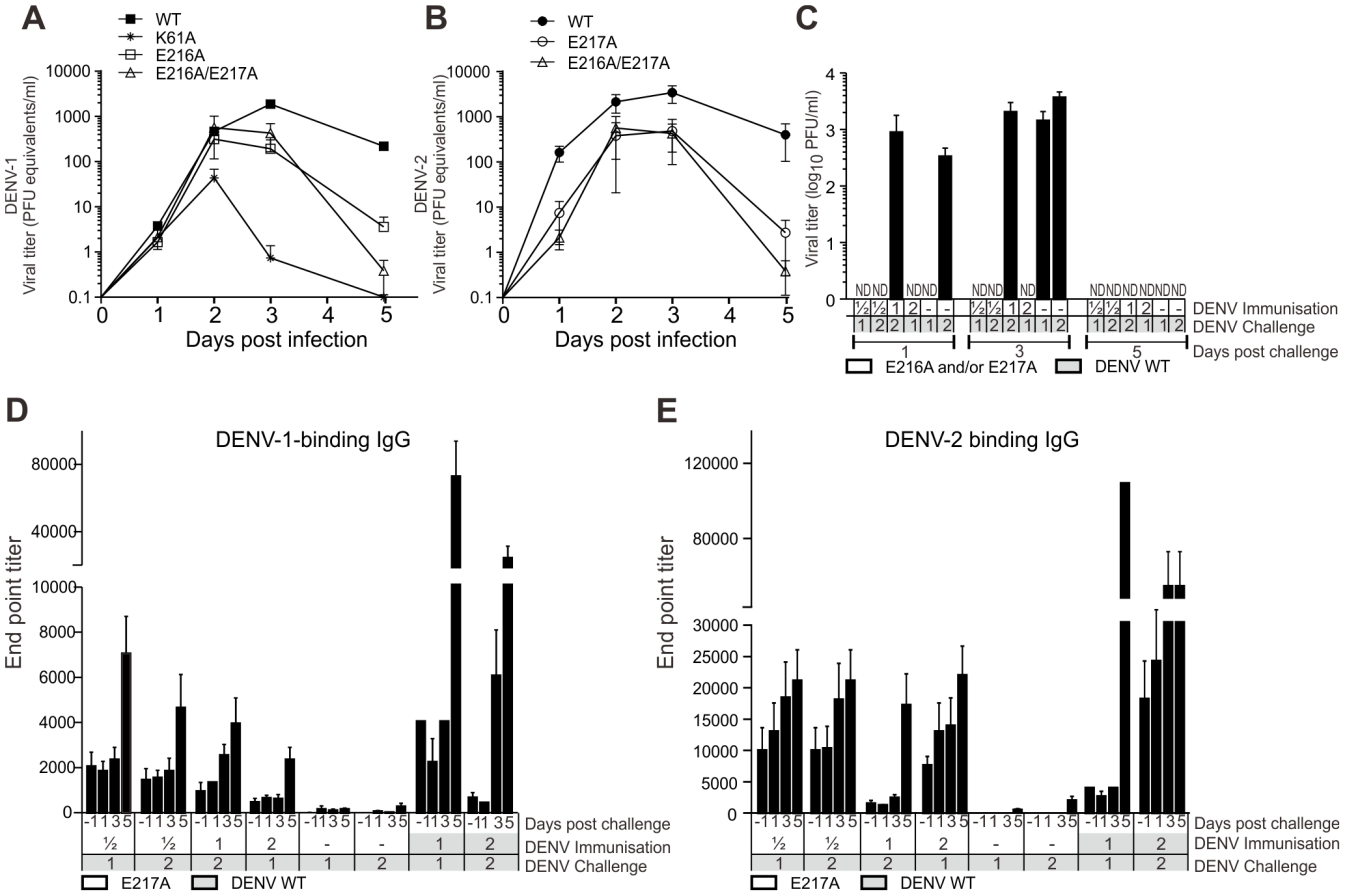
Dengue MTase mutants are attenuated and immunogenic. (A) Viremia kinetics of WT DENV-1 (strain West Pacific 74), DENV-1 K61A, and DENV-1-E216A or a combination of DENV-1 E216A and DENV-2 E217A *in vivo*. Mice were infected i.p. with 2.75×10^5^ PFU of the indicated virus. Viral titers in the plasma were measured at indicated time points by real-time RT-PCR. (B) Viral titers in plasma of mice immunized i.p. with 2.75×10^5^ PFU DENV-2 WT, DENV-2 E217A (strain TSV01) alone or in combination with DENV-1 E216A (2.75×10^5^ PFU DENV-1 E216A plus 2.75×10^5^ PFU DENV-2 E217A). Blood was taken at indicated time points and viral titers were measured by real-time PCR. (C) Viral titers in the plasma of mice immunized with 2′-*O*-MTase mutant and challenged as indicated. Numbers in gray boxes indicate WT virus, whereas numbers in white boxes indicate 2′-*O*-MTase mutant virus. Mice were immunized i.p. with 2.75×10^5^ PFU of the indicated 2′-*O*-MTase mutant serotype and challenged 30 days later with 5×10^5^ PFU WT DENV-1 strain (strain 05K3126 used for challenge due to its high virulence in mice, unpublished data) or 3×10^6^ PFU WT DENV-2. Blood was taken at indicated time points and viral titers were measured by plaque assay. ND: not detected. (D, E) IgG titers of mice immunized and challenged as described above. Blood was taken at indicated time points post challenge and IgG antibody titers against DENV-1 (D) and DENV-2 (E) were measured by ELISA. Data are representative of two experiments with three to four mice per group in each experiment (A, B) or two pooled experiments (C–E) with a total of 9 mice per group. Bars represent means with SD (A) or means with SEM (B–E).

We infected AG129 mice with the WT and 2′-*O*-MTase mutants (called “E216A” for DENV-1 and “E217A” for DENV-2 from this point) to assess viral replication and immunogenicity *in vivo*. AG129 mice lack the receptors for type I and type II IFNs, and have been used widely for antiviral and vaccine testing [25]–[28]. Mice were intraperitoneally (i.p.) infected with 2.75×10^5^ plaque-forming unit (PFU) of WT or mutant viruses. The viremia result showed that mutating K61A or E216A in DENV-1 and mutating E217A in DENV-2 attenuated the virus compared to the WT virus (Fig. 2A and B).

Next, we examined a combination of two MTase mutants (E216A and E217A) representing DENV-1 and DENV-2 to address a potential competition effect that has been described previously with attenuated strains in humans [29] and in mice [25]. To this end, mice were injected i.p. with 2.75×10^5^ PFU of E216A or E217A or a combination of both (a total of 5.5×10^5^ PFU viruses). At 30 days post immunization, mice were challenged i.p. with 1×10^6^ PFU of WT DENV-1 or 5×10^6^ WT DENV-2. DENV specific IgG titers and viremia were observed. All mice immunized with E216A and/or E217A were protected against homologous challenge (Fig. 2C), demonstrating that the immune response was protective even though the IgG titers in E216A and/or E217A-infected mice were 2 to10 times lower than those in the WT virus-infected mice (Fig. 2D and E).

A general concern for live attenuated vaccines is their theoretical potential to mutate back to WT under pressure of the immune system. To address this in our system, virus from mice infected with mutant DENV1 or DENV2 was isolated at day 3 after infection and the mutations were found to be stable (Supplementary Fig. S1c). To rule out that compensatory mutations were introduced into the viral genome the input and output (day 3 after infection) virus was sequenced using Illumina deep sequencing technology. As summarized in Supplementary Table S1, only the single nucleotide polymorphisms (SNPs) responsible for the E216A or E217A mutation were found when comparing the sequences to wild-type DENV-1 or -2, respectively.

We next compared the neutralization and infection enhancing capacity of serum collected 30 days post immunization (Table 1 and Supplementary Fig. S3) [30]. Mutant viruses cause the same or less antibody-dependent enhancement (ADE) than the respective wild-type viruses in the heterologous setting (0.51±0.16 vs. 0.74±0.2 for DENV-1 immunization and ADE tested against DENV-2 and 0.64±0.22 vs. 0.62±0.14 for DENV-2 immunization and ADE tested against DENV-1) (Table 1). More importantly, we did not observe enhanced infection *in vivo* (Fig. 2C and see later challenge experiments with a virulent DENV-2 strain). These data suggest that vaccination with the E216A/E217A mutants does not cause ADE during heterologous challenge even though lower neutralizing Ab titers are generated by the mutant strains compared to the wild-type virus.

**Table 1:** Neutralization and antibody-dependent enhancement of infection (ADE) in immunized AG129 mice.

Immunization:	NT50 (mean fold dilution ±SD)				Max. ADE (mean fold dilution±SD)			
	DENV-1	p	DENV-2	p	DENV-1	p	DENV-2	p
DENV-1 E216A	252±59		388±153	#	0.75±0.27		0.51±0.16	#
DENV-1	509±307	*	556±107		0.98±0.5	*	0.74±0.2	
DENV-2 E217A	197±188		1035±557	*	0.64±0.22		1.02±0.22	*
DENV-2	268±118		1548±566	*	0.62±0.14		1.27±0.29	*
DENV-1 E216A+DENV-2 E217A	202±78		655±261		0.94±017	*	1.05±0.32	*
PBS	88±66		251±228		0.18±0.01		0.08±0.01	

NT50 values are means ±SD of six to seven mice from two independent experiments.

Max. ADE values are normalized against 4G2, which was used as an internal standard for infection efficiency per experiment. Values are means ±SD from six to seven mice from two independent experiments.

Kruskal Wallis test with multiple comparisons:

* : p<0.05 compared to PBS.

# : p<0.05 compared to DENV-2.

### Vaccinated mice generate a non-structural protein-specific CD8 T cell response

**Figure 3:**
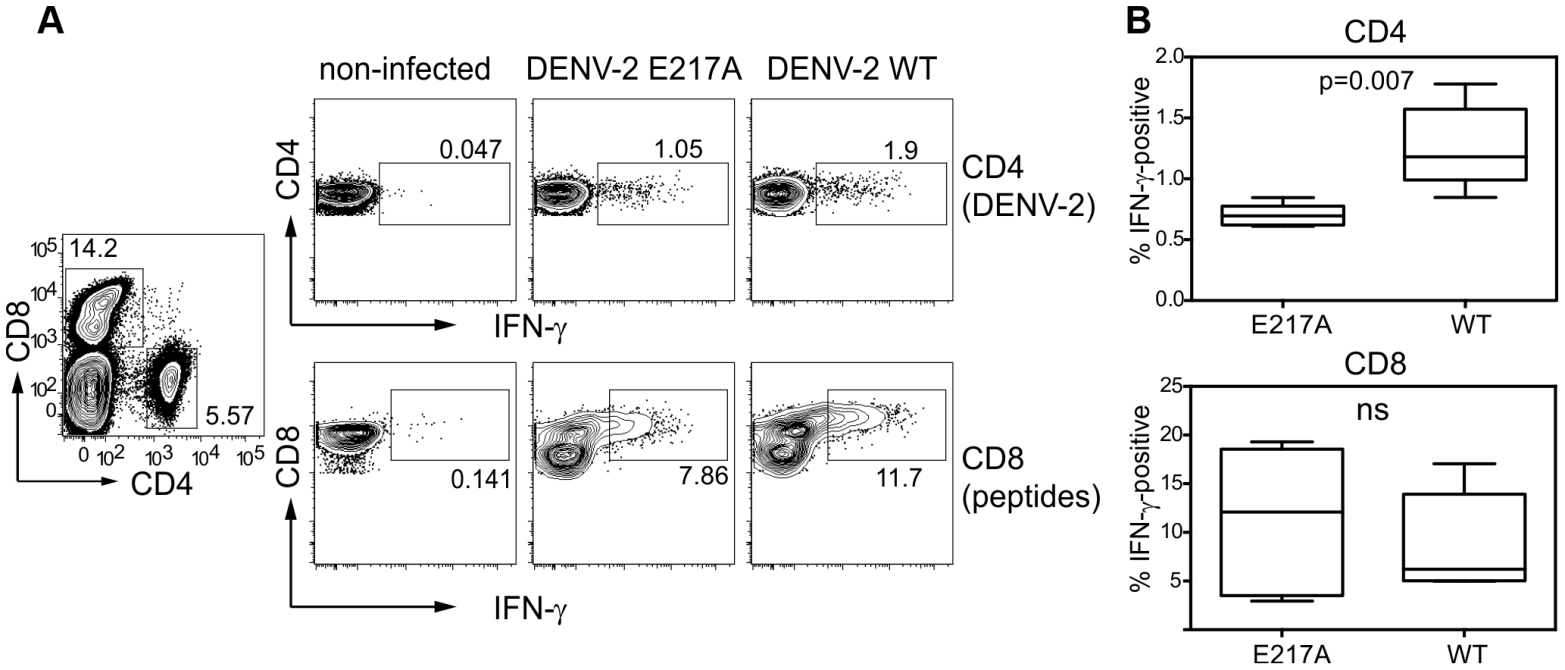
T cell IFN-γ production elicited by 2′-*O*-MTase mutant DENV-2. Splenocytes of IFNAR mice infected with DENV-2 E217A or DENV-2 WT were harvested at day 7 and were re-stimulated with DENV-2 or with peptides for the quantification of IFN-γ production in CD4 and CD8 cells. (A) Intracellular IFN-γ was measured in spleen CD4 and CD8 cells (lymphocyte gate, viable cells, cell duplets excluded) of non-immunized or immunized mice; representative graphs for each group are shown. (B) Quantitative analysis of IFN-γ production. Bars are means±SEM from two independent experiments with 2–3 mice per group in each experiment. P value was determined with a unpaired students t test.

While antibodies are crucial to reduce the viral load by binding and neutralizing virus particles, T cells are necessary for efficient viral clearance [31], [32]. AG129 mice are not suitable to study T cell responses because of their lack of IFN-γ signaling, which is critical to activate T cells. We therefore used IFNAR mice lacking the receptor for IFN-α/β [33]. IFNAR mice were immunized with 2.75×10^5^ Pfu DENV-2 E217A or DENV-2 WT and spleens were harvested at day 7 for restimulation *in vitro* and detection of IFN-γ production (Fig. 3A). Mutant and WT virus elicited a strong CD4 and CD8 T cell response after re-stimulation with DENV-2. The CD4 response was weaker in E217A-immunized mice, likely due to the lower total viral load in E217A-immunized mice compared to mice immunized with the WT virus (Fig. 3B). To test for targeted DENV T cell response splenocytes were re-stimulated with a pool of NS4B and NS5 CD8 peptides described by Yauch et al [32]. No significant difference in the NS4B and NS5-specific T cell response was seen between mice immunized with E217A or WT DENV-2 (Fig. 3B). Taken together, DENV 2′-*O*-MTase mutants induce a T cell response and epitope presentation that is similar to WT infection. Nevertheless, additional studies in mice and monkeys are necessary to assess the T cell response in greater detail and to test its functional contribution to protection.

### Vaccinated mice are protected against challenge with the virulent DENV-2 strain

**Figure 4:**
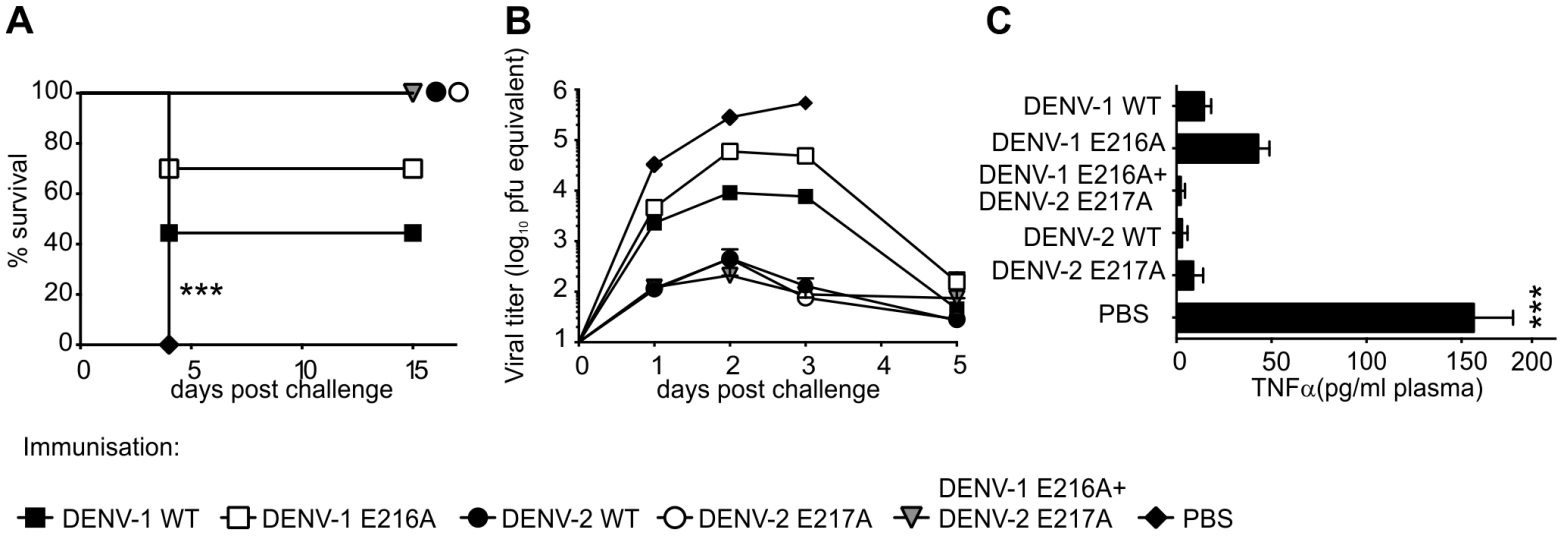
2′-*O*-MTase mutant protects against challenge with an aggressive mouse-adapted DENV strain. (A) Mice were immunized i.p. with 2.75×10^5^ DENV-1 WT, DENV-1 E216A, DENV-2 WT, DENV-2 E217A (strain TSV01) alone or in combination with DENV-1 E216A (2.75×10^5^ PFU DENV-1 E216A plus 2.75×10^5^ PFU DENV-2 E217A). 30 days post immunization mice were challenged i.p. with 10^7^ PFU of D2Y98P [34]. Health status of mice were monitored twice daily. (B) Blood was taken at indicated time points and viral titers were measured by real-time PCR. (C) TNF-α level in plasma of mice was measured at day three post challenge according to the manufacturer's protocol (eBioscience) (C). Data represent means ±SEM from 3 experiments with a total of 7–10 mice (A) or means ±SEM from two experiments with a total of 6–8 mice (B–C). Statistical analysis was performed using 1-way ANOVA Tukey's multiple comparison test (***P<.001).

DENV-1 strain 05K3126 and DENV-2 strain TSV01 do not cause pathology in mice. To test for protection against a more virulent strain we immunized mice with DENV-1 E216A, DENV-2 E217A, a mixture of E216A and E217A, WT DENV-1 (Westpac) or WT DENV-2 (TSV01) or PBS and challenged them with the virulent DENV-2 strain D2Y98P [34] 30 days later (Fig. 4). DENV-2 E217A protected against the homologous challenge (Fig. 4A). Immunization with DENV-1 E216A protected 70% of the mice, showing limited cross-protection after infection with D2Y98P (Fig. 4A and 4B). No enhanced disease was detected after heterologous challenge. Increased TNF-α levels were associated with pathology in the AG129 mouse model in the context of ADE [35]. To further assess the possibility of ADE-associated pathology, we measured TNF-α levels in plasma three days after challenge. High levels of TNF-α were only detected in unimmunized (PBS) mice, showing that TNF-α as a marker of pathology was independent of ADE, and that immunization with E216A did not cause ADE after heterologous challenge. These data demonstrate that immunization with E217A protects mice against challenge with an aggressive, virulent DENV-2 strain that causes 100% mortality in unimmunized mice.

### DENV 2′-*O*-MTase mutants are highly attenuated in macaques and induce a broad and protective immune response

To assess the safety (viremia profile) and efficacy (neutralizing antibody response and protection against challenge) of the 2′-*O*-MTase mutant DENV vaccine approach in an immunologically competent host, three groups of Rhesus monkeys (RM) were immunized with different doses of E217A. One group received a low dose (1×10^3^ PFU), one group a medium dose (1×10^4^ PFU), and one group a high dose (1×10^5^ PFU) of E217A virus. Viremia was monitored during 10 days after inoculation. The E217A virus was severely attenuated, and no viremia was detected except for one animal (R0105) that had received a high dose (1×10^5^ PFU) and developed a low viremia (Table 2). Virus was extracted for sequencing, and it was confirmed that the E217A mutation was retained in the virus extracted at days 3, 4 and 7 from this animal. Importantly, full virus genome sequencing of the viral RNA recovered at day 7 showed that no compensatory mutations were introduced (data not shown). All immunized monkeys developed neutralizing antibodies to DENV-2 on day 15 after immunization (Table 3). ADE was analyzed in a K562 assay and a similar enhancement pattern was observed for both heterologous and homologous infection *in vitro*: ADE correlated with the neutralizing titer, ie the higher the NT50 the higher the enhancement (Supplementary Fig. S4). This argues against a physiologically relevant infection enhancement, which would only be expected after heterologous infection. By day 30 after immunization, all monkeys including the ones with low dose immunization developed high titers (GMT≥92) of neutralizing antibodies (Table 3). The monkeys were then challenged with 1×10^5^ PFU of WT DENV-2 on day 64 post-immunization. No viremia was detected in all immunized monkey, whereas all four PBS-immunized controls had a mean peak virus titer of 2.5 log_10_ PFU/ml and mean viremia duration of 4.8 days (Table 4). In all animals except one (R0055), anamnestic antibody responses were observed after challenge (Table 3). These data demonstrate that live, attenuated DENV MTase mutant virus, even when administrated at low dose (1×10^3^ PFU), can induce protective immunity in non-human primates.

**Table 2:** Viremia in RMs immunized with different doses of DENV-2 E217A.

E217A dose (log10 PFU)	Monkey	Gender	Viremia (log10 PFU/ml) at indicated day post immunization										Mean	
			1	2	3	4	5	6	7	8	9	10	Peak titer (SD)	Duration days
5.0	R0319	M	0	0	0	0	0	0	0	0	0	0	0.4 (0.8)	0.8
5.0	R0212	F	0	0	0	0	0	0	0	0	0	0		
5.0	R0105	M	0	0	1.5	1.6	0	0	1.6	0	0	0		
5.0	R0942	F	0	0	0	0	0	0	0	0	0	0		
4.0	R0055	M	0	0	0	0	0	0	0	0	0	0	0	0
4.0	R0482	F	0	0	0	0	0	0	0	0	0	0		
4.0	R0098	F	0	0	0	0	0	0	0	0	0	0		
3.0	R0198	F	0	0	0	0	0	0	0	0	0	0	0	0
3.0	R0195	M	0	0	0	0	0	0	0	0	0	0		

**Table 3:** Reciprocal neutralizing antibody titer in RMs immunized with DENV-2 E217A.

E217A dose (log10 PFU)	Monkey	Gender	Reciprocal neutralizing antibody titer (PRNT50)				
			Day post immunization			Day post challenge*	
			−1	15	30	15	30
5.0	R0319	M	<10	33	106	218	597
5.0	R0212	F	<10	122	90	400	378
5.0	R0105	M	<10	55	170	339	348
5.0	R0942	F	<10	87	122	187	301
	GMT			66	119	273	392
4.0	R0055	M	<10	46	447	411	386
4.0	R0482	F	<10	31	283	400	371
4.0	R0098	F	<10	29	80	190	405
	GMT			35	216	315	387
3.0	R0198	F	<10	56	77	344	534
3.0	R0195	M	<10	17	154	597	542
3.0	R0200	F	<10	15	66	406	640
	GMT			24	92	437	570

* All animals were challenged with 1×10^5^ PFU of WT DENV-2 on day 64 post-immunization.

**Table 4:** Viremia in E217A-immunized RMs after challenge with wild-type DENV-2*.

Group (log10 PFU)	Monkey	Dose (log10 PFU)	Viremia (log10 PFU/ml) by post challenge day									Peak titer (SD)	Duration days (SD)
			1	2	3	4	5	6	7	8	9		
E217A	R0319	5	0	0	0	0	0	0	0	0	0		
5.0	R0212	5	0	0	0	0	0	0	0	0	0		
	R0105	5	0	0	0	0	0	0	0	0	0		
	R0942	5	0	0	0	0	0	0	0	0	0		
E217A	R0055	5	0	0	0	0	0	0	0	0	0		
4.0	R0482	5	0	0	0	0	0	0	0	0	0		
	R0098	5	0	0	0	0	0	0	0	0	0		
E217A	R0198	5	0	0	0	0	0	0	0	0	0		
3.0	R0195	5	0	0	0	0	0	0	0	0	0		
	R0200	5	0	0	0	0	0	0	0	0	0		
PBS	R0522	5	1.9	1.7	0	0	0	2.3	1.6	0	0	2.5(0.2)	4.8(0.5)
	R0342	5	1.6	2.8	1.7	2.4	2.1	0	0	0	0	2.5(0.2)	4.8(0.5)
	R1751	5	0	0	1.5	2.3	1.7	1.9	2.4	0	0	2.5(0.2)	4.8(0.5)
	R0351	5	0	2.0	2.0	2.6	2.4	1.6	0	0	0	2.5(0.2)	4.8(0.5)

* Animals were challenged with 1×10^5^ PFU of WT DENV-2 on day 64 post-immunization.

### IFN-β pre-treatment inhibits 2′-*O*-MTase mutant infection with the involvement of IFIT1

**Figure 5:**
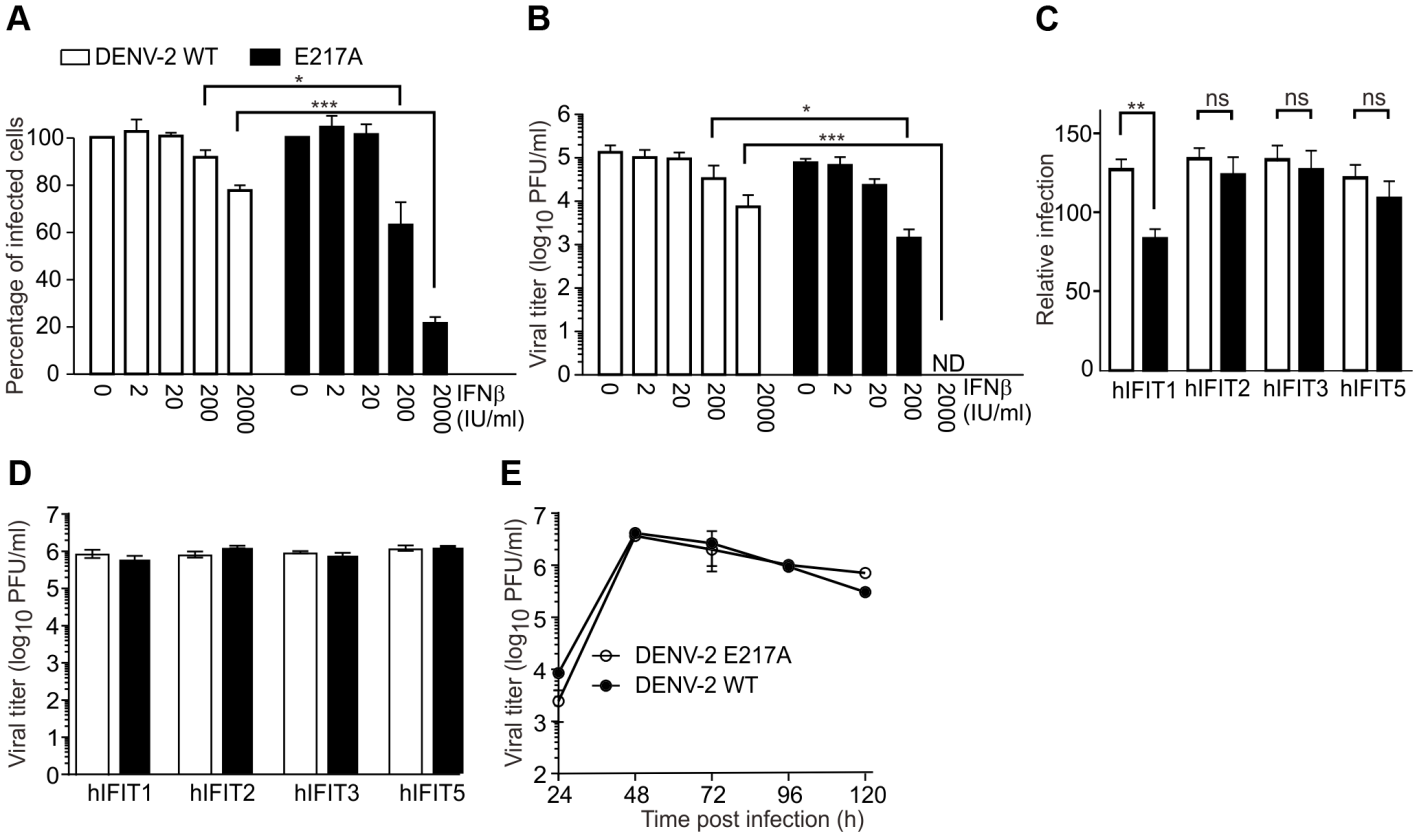
2′-*O*-MTase mutant DENV-2 has altered sensitivity to IFN, which is partially mediated by IFIT1. (A) Cells were seeded in a 24-well plate, treated for 24 h with increasing amount of IFN-β and infected with WT or E217A DENV-2. At 72 h post-infection, cells were harvested and analyzed by flow cytometry using 4G2 antibody (against viral envelope protein). (B) Viral titers in culture fluids were measured by plaque assay. Data are representative of three experiments. Means and SD are shown. Statistical analysis was performed using Student's t-test (***, p<0.001; *, p<0.05). (C) HEK293-DC-SIGN cells were transiently transfected with vector alone, human IFIT-1 (ISG56), IFIT-2 (ISG54), IFIT-3 (ISG60), or IFIT-5 (ISG58). On day 2 post-transfection, cells were infected with WT or E217A DENV-2 at an MOI of 5. The cells were analyzed for viral envelope protein expression by flow cytometry at 72 h post-infection. Results represent the mean ± SEM of six independent experiments. Percentage of infected cells was normalized to cells transfected with empty vector. (D) Virus output from transfected cells was determined in the supernatant by plaque assay. The transfection efficiency was 30–50%, (determined by parallel experiments with a GFP expression plasmid (data not shown)). (E) growth kinetics of E217A and WT DENV-2 in HEK293-DC-SIGN cells. Statistical analysis was performed using one-way ANOVA Bonferroni's multiple comparison test (**, p<0.01).

The 2′-*O*-methylation of the 5′ cap of WNV and coronavirus RNA functions to subvert innate host antiviral response through escape of IFIT-mediated suppression [14], [15]. To assess whether this is true for DENV as well, we pretreated HEK-DC-SIGN cells with an increasing dose of IFN-β for 24 h. While HEK-DC-SIGN cells are susceptible to type I IFN, they do not produce detectable levels of IFN-β after infection with mutant or WT virus (data not shown). The IFN-β-treated cells were infected with WT or mutant E217A DENV-2. The E217A virus was significantly more sensitive to IFN-β pretreatment than the WT virus, as demonstrated by the percentage of infected cells (Fig. 5A) as well as the viral titers in culture supernatants (Fig. 5B). To test the stability of the mutation under IFN pressure and in different cell types we passaged the virus in the presence of 0, 20 and 200 U/ml IFN-β in HEK-DC-SIGN and U937-DC-SIGN. As illustrated in Supplementary Fig. S5, E217A was lost in the presence of IFN, whereas wild-type virus resisted the IFN pressure in both cell lines. E217A isolated from passage three in HEK-DC-SIGN and from passage one in U937-DC-SIGN was isolated for sequencing. The E217A mutation was retained and no compensatory mutations were introduced (data not shown).

To elucidate the molecular mechanism of attenuation, we over-expressed human IFIT1, 2, 3, or 5 in HEK-DC-SIGN cells. The cells were infected with WT or mutant DENV-2 and assessed for the number of infected cells by flow cytometry (Fig. 5C). The WT virus infection was not affected, whereas E217A mutants were significantly inhibited by IFIT1, but not IFIT2, 3, or 5. However, IFIT1 over-expression did not completely block E217A infection nor did it affect virus output from the infected cells (Fig. 5D), suggesting that other IFN-mediated signals are involved in the response against DENV. Both mutant and WT virus show similar growth kinetics in untreated cells (Fig. 5E). We currently don't know why the mutant virus is attenuated in Vero cells but not in HEK-DC-SIGN since both lines are deficient in IFN production. It should be noted that the maximum antiviral effect of IFITs could be underestimated due to the low transfection efficiency (30–50%) of the IFIT-expressing plasmids.

### Inability of 2′-*O*-MTase mutant virus to infect the *Ae. aegypti* vector decreases the risk of mutant virus transmission

We compared the effect of 2′-*O*-MTase mutation on viral fitness in mosquito *Ae. aegypti*, the natural transmission vector for DENV. The mosquitoes were fed with blood containing WT or E217A. After the mosquitoes were fed at a titer of 1×10^5^ PFU/ml, significant differences in oral infection and dissemination between the WT and mutant viruses were observed 15 days post-infection (Table 5). The WT virus infected 29% of mosquitoes at the highest titer (1×10^5^ PFU/ml), but only 1–6% of mosquitoes at lower titers (1×10^3^ and 1×10^4^ PFU/ml). When orally fed with 1×10^5^ PFU/ml WT virus, approximately 10% of mosquitoes were infected after 9; the WT virus disseminated in 24% of the mosquitoes (Table 5). When fed with 1×10^3^ and 1×10^4^ PFU/ml WT virus, the dissemination rates reached 1–4%. In contrast, the mutant virus was unable to infect the *Ae. aegypti* and, subsequently, no dissemination was observed for all titers (Table 5).

**Table 5:** *Ae. aegypti* susceptibility according to virus type and titer.

Virus	Titer (log10 PFU/ml)	Infected/total female mosquitoes (%)*		?^2^	*df*	P-Value	Disseminated/total female mosquitoes# (%)		?^2^	*df*	P-Value
WT	5	24/82	(29%)	0.403	2	0.8175	20/82	(24%)	1.472	2	0.479
	4	1/72	(1%)	2.305	2	0.3159	1/72	(1%)	2.305	2	0.316
	3	3/53	(6%)	3.151	2	0.2069	2/53	(4%)	1.725	2	0.422
E217A	5	0/47	(0%)	n/a	2	n/a	0/47	(0%)	n/a	2	n/a
	4	0/40	(0%)	n/a	2	n/a	0/40	(0%)	n/a	2	n/a
	3	0/60	(0%)	n/a	2	n/a	0/60	(0%)	n/a	2	n/a

* Infected: presence of virus in abdomen.

# Disseminated: presence of virus in thorax.

To examine whether the E217A mutant could replicate *in vivo*, we intra-thoracically inoculated the WT and mutant viruses into *Ae. aegypti* mosquitoes. Intra-thoracic inoculation bypasses the mosquito midgut, which is the key barrier to establish infection during natural feeding route. Both WT and mutant viruses reached 100% infection rate upon intra-thoracic inoculation. The mean genome copy number reached 4.6×10^9^ and 6.2×10^9^, respectively (Supplementary Fig. S6). The genome copy number of the WT virus was approximately 35% higher than that of the mutant virus (p = 0.1054). Overall, the results demonstrate that the 2′-*O*-MTase mutant virus is compromised in vector fitness.

## Discussion

Various dengue vaccine strategies are currently under development, including live attenuated virus, subunit vaccines, chimeric viruses, and DNA vaccines [36], [37]. The YFV 17D-based chimeric dengue vaccine developed by Sanofi-Pasteur is the most advanced in clinical testing [38], [39]. The establishment of reverse genetic manipulation of DENV has greatly facilitated the generation of promising vaccine candidates [36], [38]. The recent progress in understanding the mechanism of attenuation of 2′-O-MTase mutant flaviviruses has provided a novel approach for vaccine and antiviral development [40]. Here we show in a proof-of-concept study that MTase mutant E216A DENV-1 and E217A DENV-2 strains are stable in vitro, and safe and immunogenic in vivo. Importantly, enhancement of infection was not observed after heterologous infection of immunized mice. The fear in a clinical setting is that sub-neutralizing titers of antibodies could enhance infections, even though this has so far not happened in the context of vaccine trials in humans [41]. A commonly used approach to address ADE *in vitro* is to infect K562 cells in the presence of antibodies. Virus alone is not able to infect K562 cells efficiently, whereas virus-antibody immune complexes bind to K562 cells via Fc-γ receptors (FcγR's), assisting the internalization of the virus and infection of the cells. We found that K562 cells could be infected in the presence of serum from immunized mice and monkeys at dilutions that were approximately 50% neutralizing in the U937-DC-SIGN system (Supplementary Fig. S3 and S4). This is in line with a previous report, which found that even strongly neutralizing antibodies are enhancing at concentrations that are close to the 50% neutralizing titer [42]. Clinically relevant ADE would be expected at sub-neutralizing titers and only after heterologous infection, and this was not observed in our experiments. A caveat of the K562 system is that the cells do not express inhibitory FcγRIIb, which is present on human target cells (dendritic cells and macrophages) and which negatively regulates ADE [43], [44]. Physiological amounts of complement, another negative regulator of ADE, are also not taken into account [45]. In summary, while the K562 assays done here did not show more ADE for heterologous infections, we cannot exclude ADE because of the limitations of the assay. Potential ADE will have to be addressed in further monkey studies.

Live attenuated dengue vaccine candidates have several advantages. Importantly, they can induce long lasting humoral and cellular immune responses to both structural and non-structural viral proteins. In this study we show a CD8 response to NS4B and NS5 peptides that is similar in mice immunized with mutant or WT virus, suggesting that the response is qualitatively equivalent. Chimeric viruses using the same backbone for all four DENV serotype glycoproteins would induce a type-specific response restricted to the structural proteins of one DENV serotype [36]. The interdependence of the T and B cell response for the efficient generation of immune memory has been demonstrated in a number of human studies [46], [47]. We speculate that the advantage of an attenuated non-chimeric DENV that includes all naturally occurring T and B cell epitopes could be that only one vaccination is required to confer long-term immunity to re-infection, as seen for natural DENV infections [48], [49]. A single-dose vaccine would facilitate the logistics of a vaccination program and would significantly reduce its cost compared to candidates requiring several booster immunizations. The 2′-*O*-MTase mutant DENV vaccine approach, with a known mechanism of attenuation, can be readily generated using a reverse genetic system. This is in contrast to the method to develop live attenuated vaccines by passaging of WT viruses in cell lines, leading to the introduction of random mutations. The reverse genetic system-based rational vaccine ensures that the vaccine maintains the attenuated genotype. Additionally, a tetravalent formulation would contain the same attenuating mutation in all four serotype recombinant vaccine strains, making the generation of a more pathogenic virus by intra vaccine-strain recombination impossible. Moreover, recombination in cell culture is hardly observed in flaviviruses, suggesting that flaviviruses are not prone to evolution by recombination [50]. By introducing additional mutations in the K-D-K-E tetrad of 2′-*O*-MTase, further safety and attenuation can be achieved. Only a virus that has at least two mutations will be acceptable in the clinical setting. Our data demonstrate that the 2′-*O*-MTase E217A virus is attenuated in mice and monkeys. We cannot explain fully why the 2′-*O*-MTase mutant virus was attenuated (10-fold lower virus titer compared to WT virus) in AG129 mice, which are unable to respond to IFN-signals. It is likely that pattern recognition receptors and downstream pathways activated by the mutant virus trigger antiviral defense mechanisms in an IFN-dependent and IFN-independent manner.

Whether the balance between low virulence and high immunogenicity is achieved in humans by 2′-*O*-MTase mutants remains to be elucidated. Our studies in human HEK293 cells show increased susceptibility of DENV2 E217A mutant to IFN-β *in vitro*, suggesting that DENV E217A mutants will be attenuated in humans as well. In the monkey immunization experiments, one monkey out of four in the high dose group experienced peak viremia of about 100 PFU, which is comparable to other live attenuated vaccine candidates [51]. Indeed, weak replication of the vaccine approach is desirable in order to induce a strong protective cellular immune response. Replication should be restricted enough to preclude onset of illness, whereas sub-clinical symptoms such as mild rash, transient leukopenia, and mildly elevated liver enzyme values are generally accepted [52]–[54]. Furthermore, studies with murine hepatitis virus have shown that MTase mutants are highly attenuated in its natural host, induce IFN, which could further induce the immunogenicity of a vaccine, and are genetically stable *in vivo* [15]. Moreover, the replication level of WNV 2′-*O*-MTase mutant in mice was largely decreased in the spleen, serum, or brain in comparison with the WT WNV infection. Intracranial inoculation of 1×10^5^ PFU of 2′-*O*-MTase mutant WNV did not cause any mortality and morbidity in mice, demonstrating the safety of this vaccine approach [14]. Taken together, these evidences demonstrate the safety and immunogenicity of the MTase-mutant vaccine approach. We are currently working on the tetravalent formulation to develop the strategy towards a clinical application.

## Materials and Methods

### Ethics statement

All experimental procedures involving Rhesus Monkeys were approved by and carried out in strict accordance with the guidelines of the Animal Experiment Committee of State Key Laboratory of Pathogen and Biosecurity, Beijing, China. All procedures were performed under sodium pentobarbital anesthesia by trained technicians and all efforts were made to ameliorate the welfare and to minimize animal suffering in accordance with the “Weatherall report for the use of non-human primates” recommendations.

The mouse experiments were conducted according to the rules and guidelines of the Agri-Food and Veterinary Authority (AVA) and the National Advisory Committee for Laboratory Animal Research (NACLAR), Singapore. The experiments were reviewed and approved by the Institutional review board of Biological Resource Center, Singapore (IACUC protocols 90474 and 100536).

### Cells

BHK-21, C6/36, and HEK-293 were purchased from the American type culture collection (http://www.atcc.org). HEK-293 and U937 cells expressing DC-SIGN were obtained by lentiviral transfection and subsequent cell sorting. All cells were maintained in minimal essential medium supplemented with fetal bovine serum (5%–10%).

### Recombinant MTase preparation and methylation assays

WT MTases representing the N-terminal 262 and 296 amino acids of DENV-1 and -2 NS5, respectively, were cloned, expressed, and purified as reported previously [11]. Mutagenesis of MTase was performed using QuikChange II XL site-directed mutagenesis kit (Stratagene). The complete sequence of each mutant MTase was verified by DNA sequencing. N7- and 2′-O-methylation assays were performed as described before [11].

### Preparation and characterization of recombinant DENV

Full-length infectious cDNA clones of DENV-1 (Western Pacific 74 strain) and DENV-2 (TSV01 strain) [55], [56] were used to generate WT and mutant viruses. A standard mutagenesis protocol was used to engineer mutations into the MTase region as reported previously [11]. The protocols for in vitro transcription, RNA transfection, IFA, plaque assay, and growth kinetics were reported previously [23]. Strain D2Y98P was described previously [34].

### Mice

Female or male 6–8 week old IFN α/β/γ receptor deficient mice (AG129) were purchased from B&K Universal Limited with permission from Dr. M. Aguet (ISREC, School of Life Sciences Ecole Polytechnique Fédérale (EPFL)). IFN α/β receptor deficient mice (IFNAR) on a C57BL/6 background were provided by Prof. Ulrich Kalinke [33]. All mice were bred and kept under specific pathogen-free conditions at the Biomedical Resource Centre, Singapore. For immunization, BHK-21 derived mutant and WT viruses were used. For challenge experiments DENV produced in C6/36 cells was used.

### Rhesus monkey study

Fourteen RMs, weighing from 3.4 to 5.0 kg, were pre-screened negative for IgG antibodies against DENV and JEV by indirect immunofluorescence assay. Animals were randomly divided into four groups and inoculated subcutaneously (s.c.) in the deltoid region of left arm with 0.5 ml of DENV-2 E217A dilutions containing 5.0, 4.0, 3.0 log10 PFU, respectively. Animals in the control group received PBS. Blood was collected from each RM daily post immunization for 10 days to detect viremia. For neutralizing antibody tests, blood was taken before immunization (day −1) and on days 15 and 30 post-immunization. On day 64 post-immunization, all immunized animals including the PBS-treated control animals were challenged by s.c. inoculation of 0.5 ml containing 5 log_10_ PFU of DENV-2 (TSV-01). For the following 9 days, blood was collected for determination of viremia. Neutralizing antibody levels in serum were measured by the standard 50% plaque reduction neutralization test (PRNT50) on days 15 and 30 post-challenge, respectively.

### Determination of viremia in monkey sera

Viremia in serum samples was determined by plaque assay in BHK-21 cell monolayers in 12-well plates. Undiluted serum or serial 10-fold dilutions of serum were inoculated onto BHK cells. After 1 h of adsorption at 37°C, wells were overlaid with 1 ml of DMEM supplemented with 2% FBS and 1% agarose. Plates were incubated for 4 days at 37°C in 5% CO_2_. Monolayers were fixed by addition of 1 ml of 4% formalin solution to the overlay medium. After 1 h of fixation at room temperature, the fixative was removed, wells were washed with water, and monolayers were stained with 1% crystal violet in 70% methanol. Plaques were counted, and titers were expressed as PFU/ml.

### Plaque reduction neutralization test

For determination of dengue virus-neutralizing antibody titers, serial twofold dilutions of serum (starting at a dilution of 1∶10) were mixed with equal volumes of a suspension of ∼500 PFU of DENV-2 TSV01/ml. The serum-virus mixtures were incubated at 37°C for 1 h and tested (0.2 ml/well) for concentration of infectious virus using the plaque assay described above. The neutralization titer was defined as the lowest serum dilution at which the infectious virus concentration was reduced by 50% from the concentration found when virus was incubated with culture medium.

### IFN pretreatment and IFIT overexpression

Cells were seeded at 1×10^5^ per well in a 24-well plate and treated 24 hours prior to infection with medium or varying concentrations of human recombinant IFN-β (Immunotools). Cells were then infected at an MOI of 1 with WT or MTase mutant virus (TSV01), respectively, incubated for 72 hours and harvested and processed for flow cytometry as described. Supernatants were collected for plaque assay.

IFIT expression plasmids were a kind gift from A. Pichlmair (14). For IFIT overexpression cells were seeded at 1×10^6^ per well in a 6-well plate. 24 hours later cells were transfected using 293fectin according to manufacturer's protocol. One day post transfection cells were trypsinized and seeded in a 24-well plate at 1×10^5^ per well. After 24 hours of incubation cells were infected and analysed as described previously. Transfection rate was 30–50% judged by parallel experiments with GFP expression plasmid.

### Detection of infection by flow cytometry, ADE assay and flow cytometry-based neutralization assay

To determine the percentage of infected cells, cells were harvested, washed in PBS and fixed and permeabilized with Cytofix/Cytoperm (BD). Intracellular dengue E protein was stained with antibody 4G2 conjugated to Alexa 647 and fluorescent cells were measured by flow cytometry.

For the assessment of ADE, 4G2 or serum/plasma was serially diluted and a constant amount of virus was added. The antibody-virus mixture was incubated at 37°C for 30 min and then 50 µl of the mixture was added to 25'000 K562 cells per 96-plate well (MOI 0.5–1). After 2 h of infection 150 µl RPMI medium containing FCS was added. After 2.5 days of incubation the infected cells were fixed and stained intracellularly with 4G2-Alexa 647. The percentage of infected cells was quantified by flow cytometry.

For the measurement of neutralization, 4G2 or heat-inactivated serum/plasma was serially diluted and a constant amount of virus was added. The antibody-virus mixture was incubated at 37°C for 30 min and then 50 µl of the mixture was added to 200,000 U937 cells (ATCC) stably transfected with human DC-SIGN (MOI 0.1–1). After 2 h of infection 150 µl RPMI medium containing FCS was added. After incubation over night the infected cells were fixed and stained intracellularly with 4G2-Alexa 647. The percentage of infected cells was quantified by flow cytometry and data were analyzed with GraphPad Prism software for the calculation of the NT50.

### T cell re-stimulation

Spleens were harvested at day 7 after infection and single cell suspensions were incubated with live virus or a pool of the following peptides: NS4B_59-66_, NS4B_99-107_ and NS5_237-245_ [32] overnight. Brefeldin (Biolegend) was added for 5 h before cells were washed and stained with antibodies CD4-APC, CD8-PercPCy5.5 and IFN-γ-PE-Cy7 (Biolegend). Cells were acquired on a FACSCantoII (BectonDickinson) and data were analyzed with FlowJo (Treestar ltd.)

### IgG ELISA

96-well polystyrene plates were coated with concentrated, heat inactivated dengue virus. Plates were incubated overnight at 4°. Before use, plates were washed three times in PBS (pH 7.2) containing 0.05% Tween-20 (PBS-T). Non-specific binding was blocked with 2% non-fat dry milk diluted in PBS (PBS-M) for 2 h at room temperature (RT). After washing, sera were diluted 1∶50 in PBS-M, heat inactivated for 1 hour at 55°C and threefold serial dilutions were added to the wells. Plates were incubated for 1 h at RT, followed by three washes with PBS-T. Peroxidase-conjugated rabbit anti-mouse IgG, in PBS-M was added, followed by 1 h of incubation at RT and three additional washes with PBS-T. TMB was used as the enzyme substrate. The reaction was stopped with 1 M HCl and the optical densities were read at 450 nm using an automatic ELISA plate reader. Endpoint titers were defined as the lowest dilution of plasma in which binding was twofold greater than the mean binding observed with the negative controls.

### Vector competence experiments

Vector competence experiments were performed using a colony of Ae. aegypti mosquitoes in which 10% of the population is derived from field obtained eggs each month. Batches of 50–75 female mosquitoes, aged 5–7 days were fed with pig blood containing WT or MTase mutant DENV-2 at titers of 5, 4, and 3 log 10 PFU/ml. Fully engorged mosquitoes were held at 27°C, 80% relative humidity, and 12 h photoperiod for 15 days, after which the abdomen was separated from the thorax and homogenized. Homogenates were inoculated into Vero cell culture. After culturing the inoculated cells for 5 days, viral infection was assayed using an indirect fluorescent antibody test (IFA). Antibody 6B6C-1 against flavivirus group E protein (at 1∶10 dilution; provided by the USA CDC as a mouse hybridoma) and an anti-mouse antibody conjugated with FITC were used as a primary and secondary antibody, respectively. Positive fluorescence determinations were performed manually using an inverted fluorescent microscope (Olympus IX71). Chi-square and contingency table statistical tests were performed to detail heterogeneity in vector competence within/between WT and mutant viruses.

Intra-thoracic inoculation of 0.17 µl of WT DENV-2 and E217A at a titer of 10^5^ PFU/ml was performed using 10 female mosquitoes each. Following inoculation, mosquitoes were held for seven days under the same conditions as described above. Mosquitoes were then killed by freezing and homogenized. Viral RNA was quantified by real-time qRT-PCR using primers and methods reported previously [57]. Briefly, whole mosquito homogenate viral RNA was extracted using QIAamp Viral RNA Mini Kit (Qiagen). qRT-PCR was completed using Invitrogen SuperScript III Platinum One-Step qRT-PCR mix (without ROX) and CFX96 Real-Time PCR Detection System (BioRad). Cycling parameters performed were 50°C for 30 min, 95°C for 2 min, followed by 45 cycles of 95°C for 10 sec, 60°C for 30 sec. A two-tailed unpaired t-test was performed to determine the statistic difference between the mean genomic equivalents calculated for WT and mutant viruses.

### Deep sequencing

Virus was isolated from mouse serum with Qiagen Viral RNA extraction Kit.

Fifty ng of viral RNA were used to prepare cDNA libraries using the Illumina TruSeq RNA sample preparation kit according to manufacturer's protocol. The only protocol modification was the removal of the mRNA enrichment step. The cDNA libraries were sequenced as a multiplex in a single lane of an Illumina HiSeq2000 (Next Generation Sequencing Core facility, Genomic Institute of Singapore). One to 2 million 50 bp paired-end reads were generated for each virus.

Wild-type and mutant virus samples were mapped to their respective reference genomes using Bowtie 2 [58]. Mapping statistics and genotype calls were made with SAMtools [59]. Data analysis was performed in Pipeline Pilot (http://www.accelrys.com). At least two reads with an alternate base at a given position were defined as a SNP.

### Statistical analysis

Statisitical tests were performed with GraphPad Prism software, using students t test, two-way ANOVA or Chi-square and contingency table statistical tests as indicated in the figure legends.

## Supporting Information

Figure S1 **Genetic stability of the E216/E217A mutation in vitro and in vivo.** Indicated mutant virus were passaged 10 times on Vero cells (a) or HEK-DC-SIGN cells (b). c) Mice were infected with 2.75×10^5^ PFU of the indicated virus and viral RNA was isolated from plasma three days post infection. Shown are sequences of RT-PCR products from the mutated region. The mutation sites are indicated with red boxes.
(TIF)

Figure S2 **Characterization of DENV-1 MTase.** (a) SDS-PAGE analysis. DENV-1 and DENV-2 MTases were expressed and purified [23]. The recombinant proteins were analyzed on a 12% SDS-PAGE. DENV-1 and DENV-2 MTases contained the N-terminal 262 and 296 amino acids of NS5 protein, respectively. Molecular masses of protein markers are labeled. Note that amino acid E216 of DENV-1 MTase is equivalent to amino acid E217 of DENV-2-MTase. (b) Effects of E216A and K61+E216A mutations of MTase on N7- and 2′-*O* methylation activities. Relative methylation activities were indicated below the TLC images with WT activity set as 100%. (c) Immunofluorescence analysis (IFA). BHK-21 cells were transfected with equal amounts of WT and mutant genome-length RNAs of DENV-2. The cells were examined for viral E protein expression at indicated days post transfection. (d) Plaque morphology. WT and mutant DENV-1 recovered from viral RNA-transfected cells (passage 0), as well as the viruses after culturing on Vero cells for 10 rounds (passage 10) were analyzed by plaque assays. (e) Growth kinetics. Vero and C3/36 cells were infected with WT and mutant DENV-1 at an MOI of 0.1, and measured for viral yields at indicated time points. Average results of three experiments are presented.
(TIF)

Figure S3 **Neutralization and ADE assay with AG129 mouse plasma.** Plasma from AG129 mice was analyzed 30 days after immunization with mutant or wild-type DENV. Upper graphs in panels a, b and c show ADE assays using K562 cells and lower graphs show the corresponding neutralization assay using U937-DC-SIGN as target cells. Groups of mice were immunized with a) DENV-1 E216A, DENV-1 WT, DENV-1 E216A and DENV-2 E217A combined or PBS; b) DENV-2 E217A or DENV-2 WT. C) Antibody 4G2 was used as a technical control. Symbols in panels a) and b) are the means ±SEM of three mice per group, tested in duplicate. The shown experiment is representative for one of two. The mean ±SD from the two independent experiments (n = 3–4 per group) are shown in Table 1.
(TIF)

Figure S4 **Neutralization and ADE assay with NHP serum.** The serum of three monkeys per group was analyzed for ADE activity. Sera from day 5 after challenge in PBS animals (day 5 post infection) or 5 days after challenge in animals which had been immunized with E217A 64 days earlier (day 5 post challenge). a) K562 cells were infected with DENV-1 or DENV-2 in the presence of serum diluted as indicated in the x axes. Symbols are means±SEM of three sera per group from two independent ADE assays testing the sera in duplicate each. b) The same sera were tested for neutralization by using U937-DC-SIGN as target cells. Symbols are means±SD of three sera per group, tested in duplicate each. c and d) 4G2 antibody was used as a technical control for the infection of K562 cells (c) or U937-DC-SIGN cells (d). Symbols are means±SD of duplicate values.
(TIF)

Figure S5 **E217A does not mutate and escape IFN pressure in human cell lines HEK293-DC-SIGN and U937-DC-SIGN.** HEK293-DC-SIGN cells (a) and U937-DC-SIGN cells (b) were seeded in a 24well plate, incubated for 24 hours with 0, 20 or 200 IU/ml of IFN-β and infected at MOI of 1 with E217A or WT DENV-2. 48 hours post infection the percentage of infected cells was determined by FACS. 100 µl of the supernatant (passage p1) was transferred to newly seeded IFN-β pre-treated cells. The remaining supernatant was kept for isolation of viral RNA and sequencing. This procedure was repeated two more times (p2 and p3). P3 was collected after 96 instead of 48 hours to allow any potential mutants to have enough time to grow to high titers.
(TIF)

Figure S6 **Comparison of genome copy numbers of mutant (MT) and wild-type (WT) virus after intra-thoracic infection of** ***Ae. aegypti*** **.** Ten female mosquitoes were inoculated intra-thoracic with 0.17 µl of WT DENV-2 or E217A at a titer of 10^5^ PFU/ml. Seven days later mosquitoes were killed by freezing and homogenized. Viral RNA was quantified by real-time qRT-PCR. Mean and 95% CI intervals are indicated by horizontal bars, each point represents a single female mosquito. P = 0.105, unpaired t test.
(TIF)

Table S1 **SNPs of virus recovered from mice at day 3 after infection.**
(DOCX)
