# Correction: Rational Design of a Live Attenuated Dengue Vaccine: 2′-*O*-Methyltransferase Mutants Are Highly Attenuated and Immunogenic in Mice and Macaques

**DOI:** 10.1371/annotation/4d0a4eb9-24be-4d26-bef0-c6cdc8824c69

**Published:** 2013-09-10

**Authors:** Roland Züst, Hongping Dong, Xiao-Feng Li, David C. Chang, Bo Zhang, Thavamalar Balakrishnan, Ying-Xiu Toh, Tao Jiang, Shi-Hua Li, Yong-Qiang Deng, Brett R. Ellis, Esther M. Ellis, Michael Poidinger, Francesca Zolezzi, Cheng-Feng Qin, Pei-Yong Shi, Katja Fink

The following sentence was missing from the Funding section: PYS is partially supported by Singapore STOPDengue TCR grant.

