# Supplementary figures and images for: Rational Design of a Live Attenuated Dengue Vaccine: 2′-O-Methyltransferase Mutants Are Highly Attenuated and Immunogenic in Mice and Macaques
Source: PLoS Pathog. 2013 Aug 1;9(8):e1003521. doi: 10.1371/journal.ppat.1003521 (PMC3731252; doi:10.1371/journal.ppat.1003521)

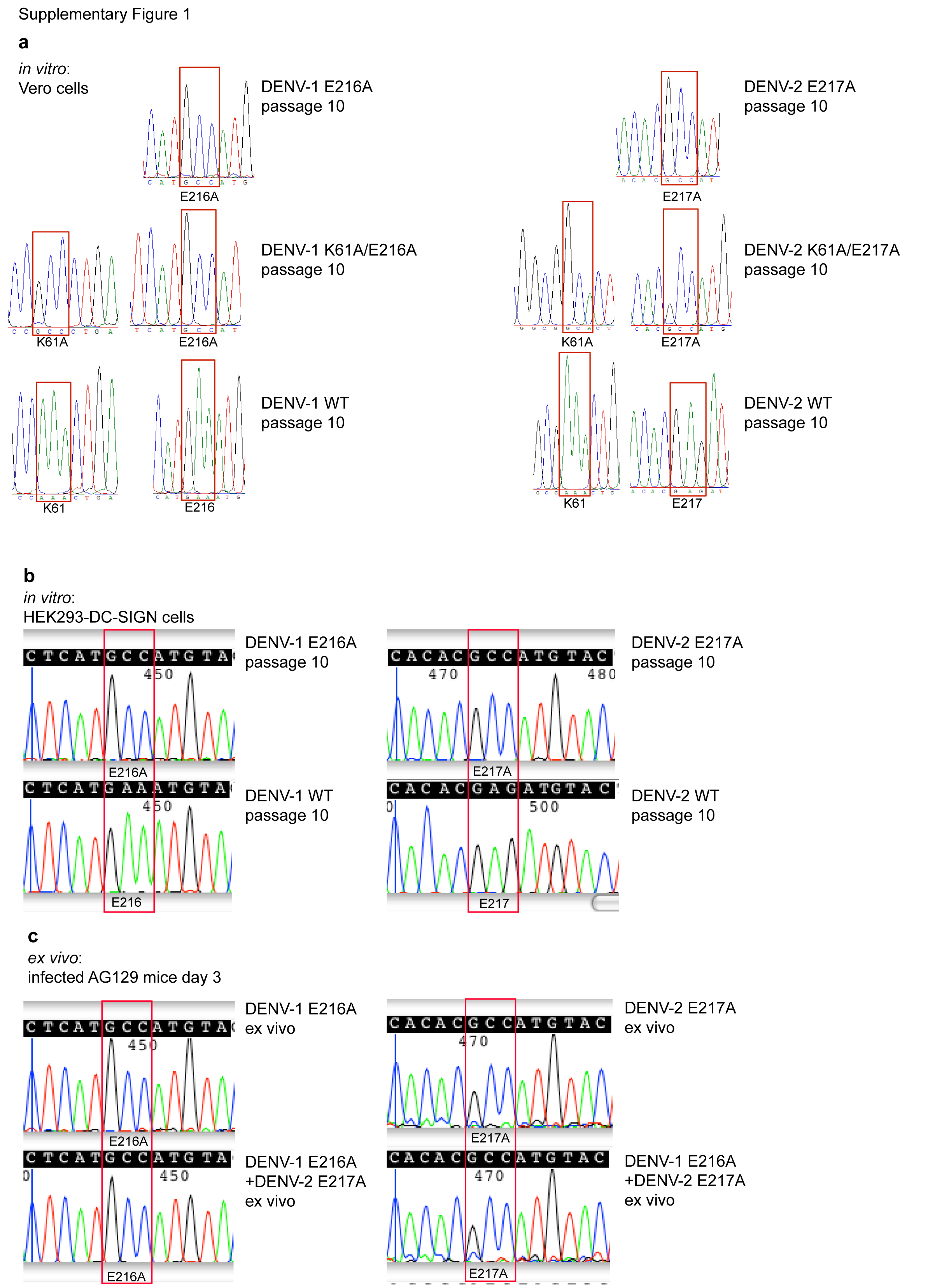

Supplement: Figure S1 — Genetic stability of the E216/E217A mutation in vitro and in vivo. Indicated mutant virus were passaged 10 times on Vero cells (a) or HEK-DC-SIGN cells (b). c) Mice were infected with 2.75×10^5^ PFU of the indicated virus and viral RNA was isolated from plasma three days post infection. Shown are sequences of RT-PCR products from the mutated region. The mutation sites are indicated with red boxes. (TIF) [file ppat.1003521.s001.tif]

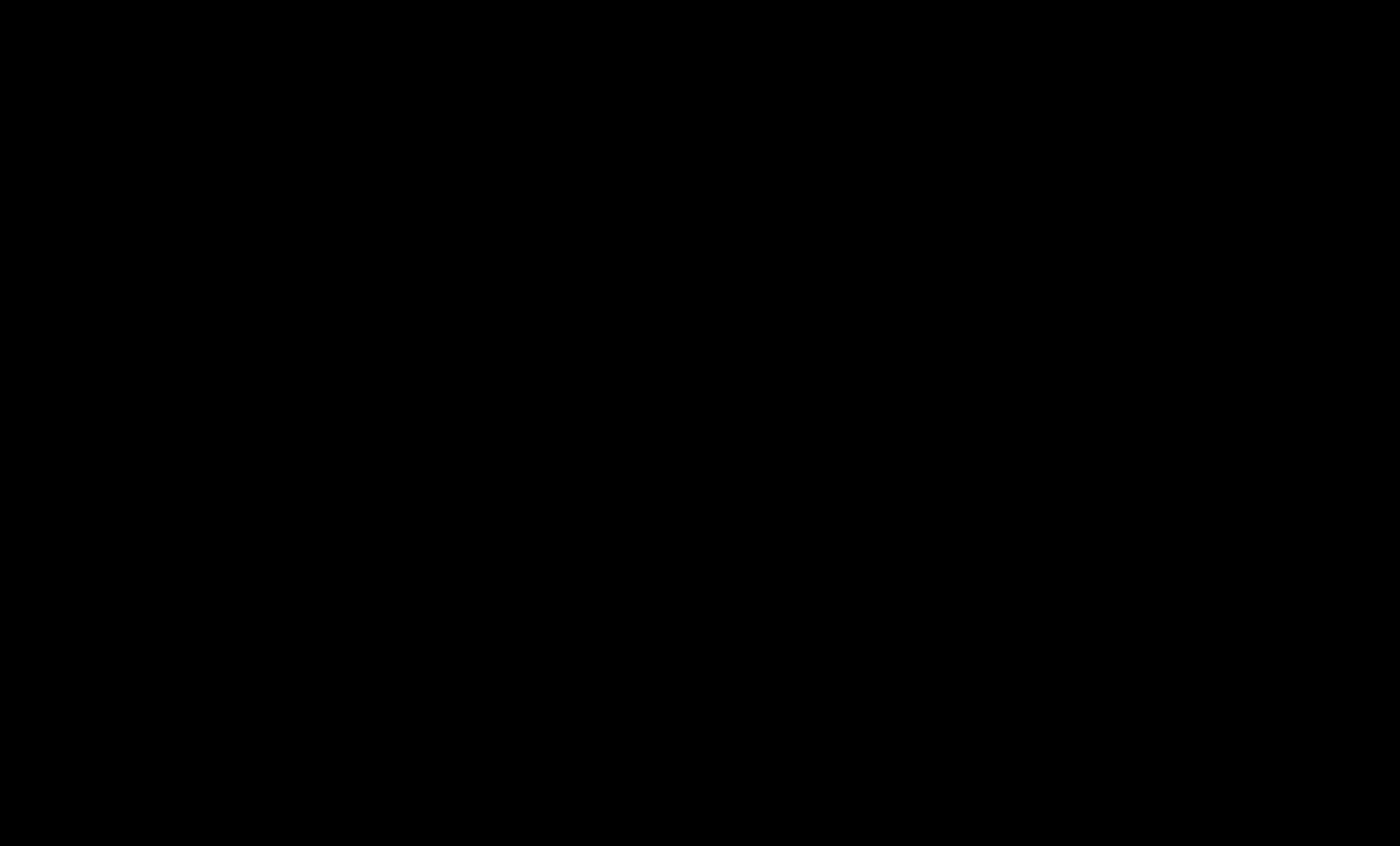

Supplement: Figure S2 — Characterization of DENV-1 MTase. (a) SDS-PAGE analysis. DENV-1 and DENV-2 MTases were expressed and purified [23]. The recombinant proteins were analyzed on a 12% SDS-PAGE. DENV-1 and DENV-2 MTases contained the N-terminal 262 and 296 amino acids of NS5 protein, respectively. Molecular masses of protein markers are labeled. Note that amino acid E216 of DENV-1 MTase is equivalent to amino acid E217 of DENV-2-MTase. (b) Effects of E216A and K61+E216A mutations of MTase on N7- and 2′-O methylation activities. Relative methylation activities were indicated below the TLC images with WT activity set as 100%. (c) Immunofluorescence analysis (IFA). BHK-21 cells were transfected with equal amounts of WT and mutant genome-length RNAs of DENV-2. The cells were examined for viral E protein expression at indicated days post transfection. (d) Plaque morphology. WT and mutant DENV-1 recovered from viral RNA-transfected cells (passage 0), as well as the viruses after culturing on Vero cells for 10 rounds (passage 10) were analyzed by plaque assays. (e) Growth kinetics. Vero and C3/36 cells were infected with WT and mutant DENV-1 at an MOI of 0.1, and measured for viral yields at indicated time points. Average results of three experiments are presented. (TIF) [file ppat.1003521.s002.tif]

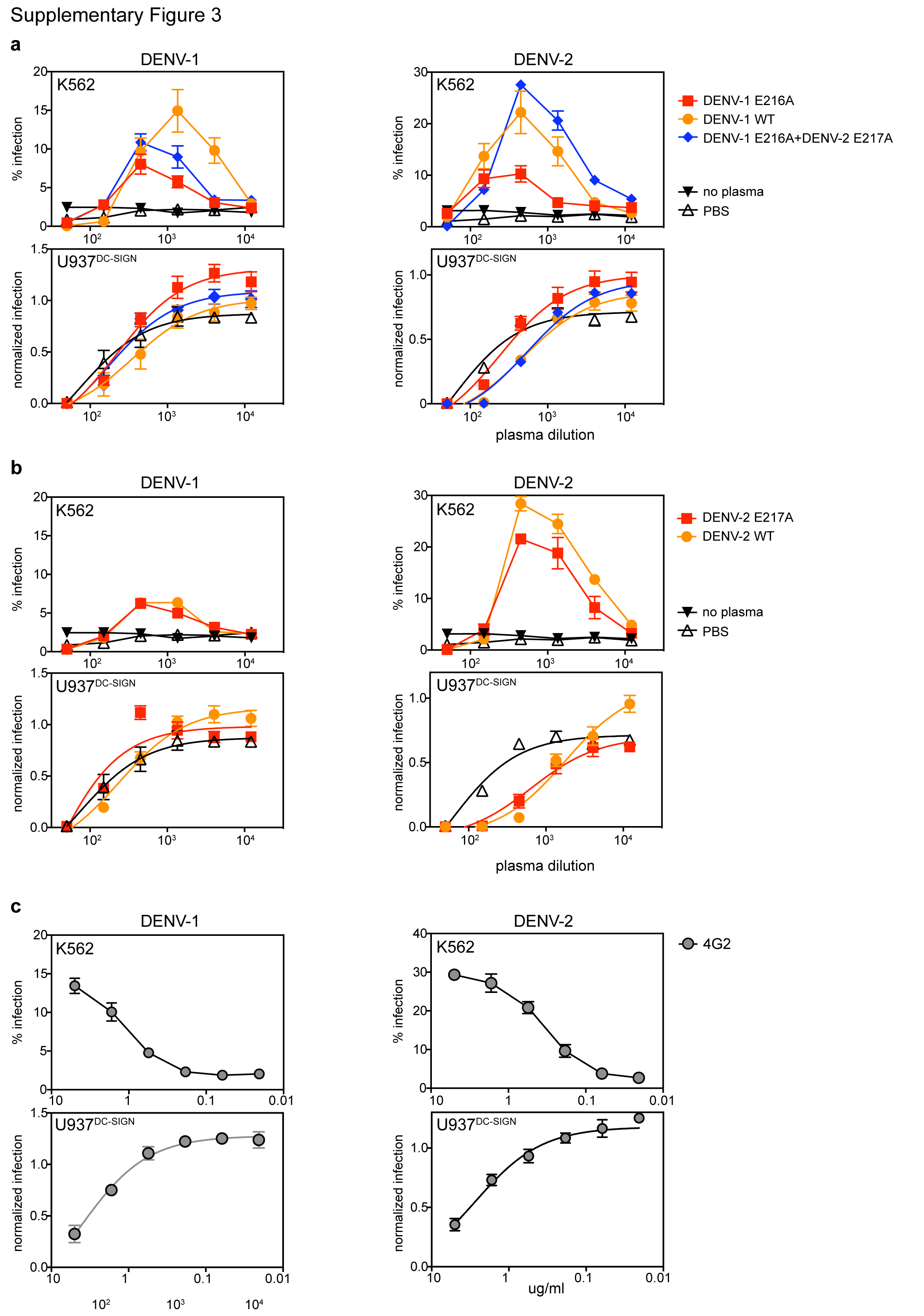

Supplement: Figure S3 — Neutralization and ADE assay with AG129 mouse plasma. Plasma from AG129 mice was analyzed 30 days after immunization with mutant or wild-type DENV. Upper graphs in panels a, b and c show ADE assays using K562 cells and lower graphs show the corresponding neutralization assay using U937-DC-SIGN as target cells. Groups of mice were immunized with a) DENV-1 E216A, DENV-1 WT, DENV-1 E216A and DENV-2 E217A combined or PBS; b) DENV-2 E217A or DENV-2 WT. C) Antibody 4G2 was used as a technical control. Symbols in panels a) and b) are the means ±SEM of three mice per group, tested in duplicate. The shown experiment is representative for one of two. The mean ±SD from the two independent experiments (n = 3–4 per group) are shown in Table 1. (TIF) [file ppat.1003521.s003.tif]

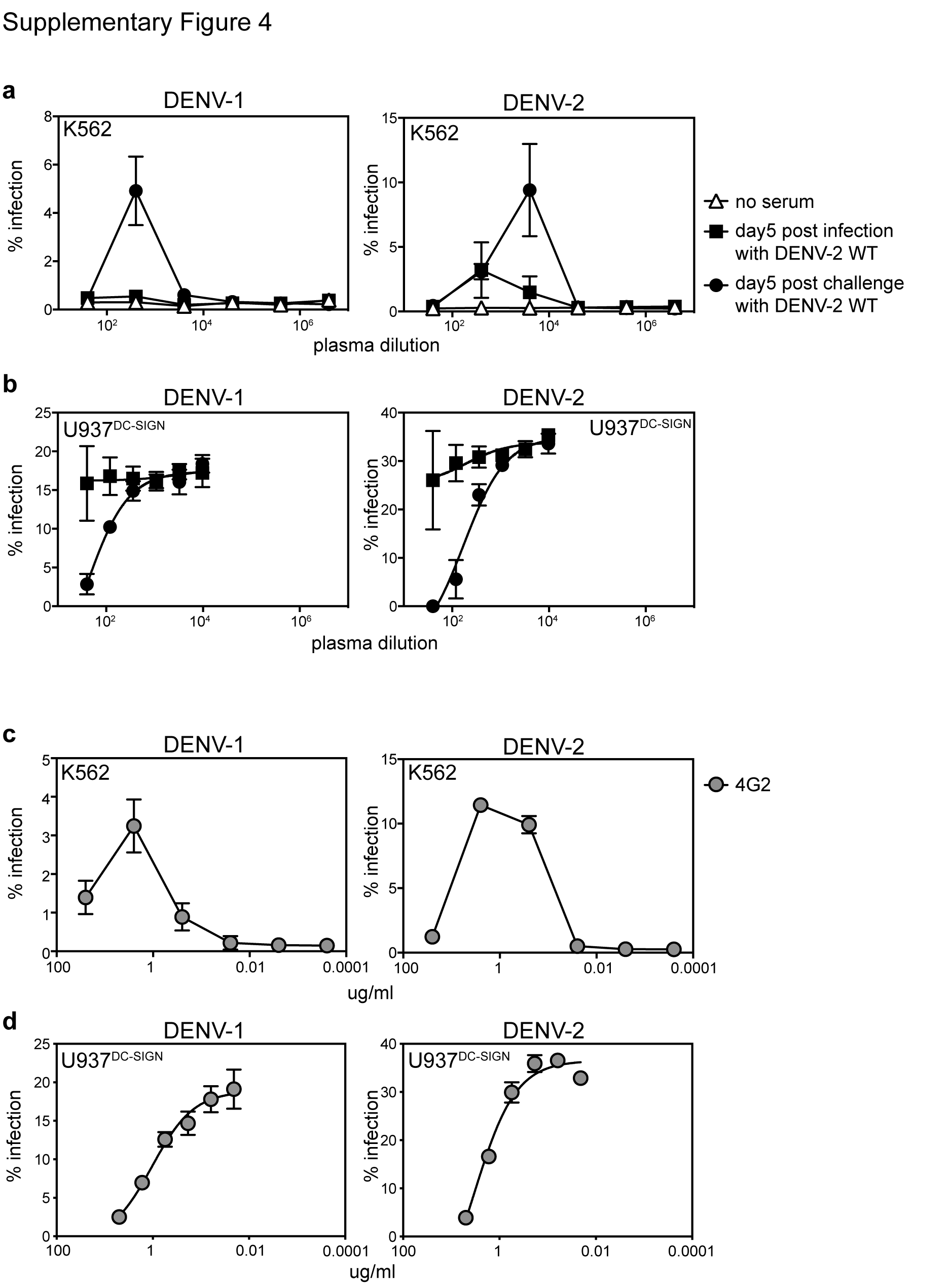

Supplement: Figure S4 — Neutralization and ADE assay with NHP serum. The serum of three monkeys per group was analyzed for ADE activity. Sera from day 5 after challenge in PBS animals (day 5 post infection) or 5 days after challenge in animals which had been immunized with E217A 64 days earlier (day 5 post challenge). a) K562 cells were infected with DENV-1 or DENV-2 in the presence of serum diluted as indicated in the x axes. Symbols are means±SEM of three sera per group from two independent ADE assays testing the sera in duplicate each. b) The same sera were tested for neutralization by using U937-DC-SIGN as target cells. Symbols are means±SD of three sera per group, tested in duplicate each. c and d) 4G2 antibody was used as a technical control for the infection of K562 cells (c) or U937-DC-SIGN cells (d). Symbols are means±SD of duplicate values. (TIF) [file ppat.1003521.s004.tif]

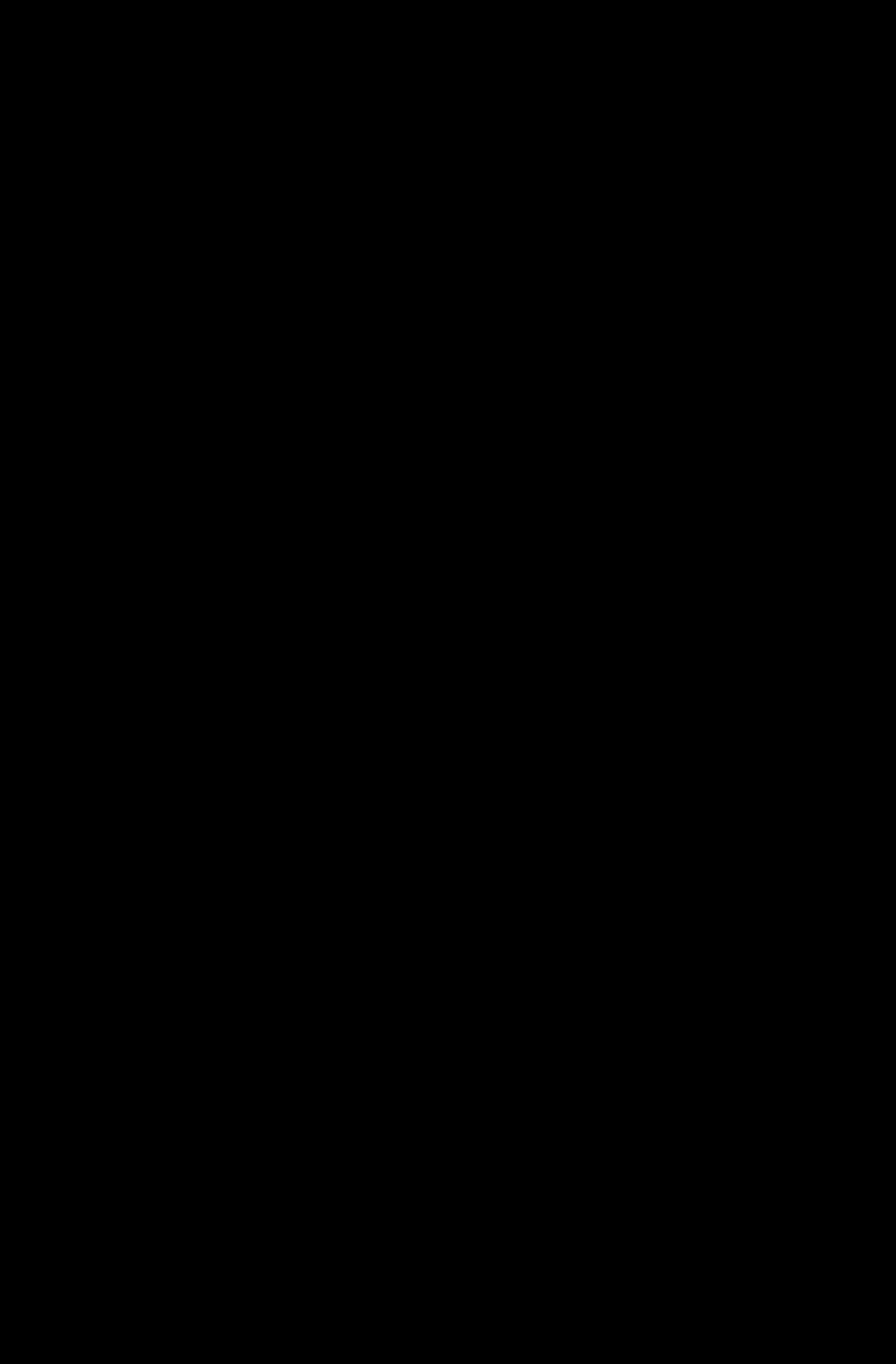

Supplement: Figure S5 — E217A does not mutate and escape IFN pressure in human cell lines HEK293-DC-SIGN and U937-DC-SIGN. HEK293-DC-SIGN cells (a) and U937-DC-SIGN cells (b) were seeded in a 24well plate, incubated for 24 hours with 0, 20 or 200 IU/ml of IFN-β and infected at MOI of 1 with E217A or WT DENV-2. 48 hours post infection the percentage of infected cells was determined by FACS. 100 µl of the supernatant (passage p1) was transferred to newly seeded IFN-β pre-treated cells. The remaining supernatant was kept for isolation of viral RNA and sequencing. This procedure was repeated two more times (p2 and p3). P3 was collected after 96 instead of 48 hours to allow any potential mutants to have enough time to grow to high titers. (TIF) [file ppat.1003521.s005.tif]

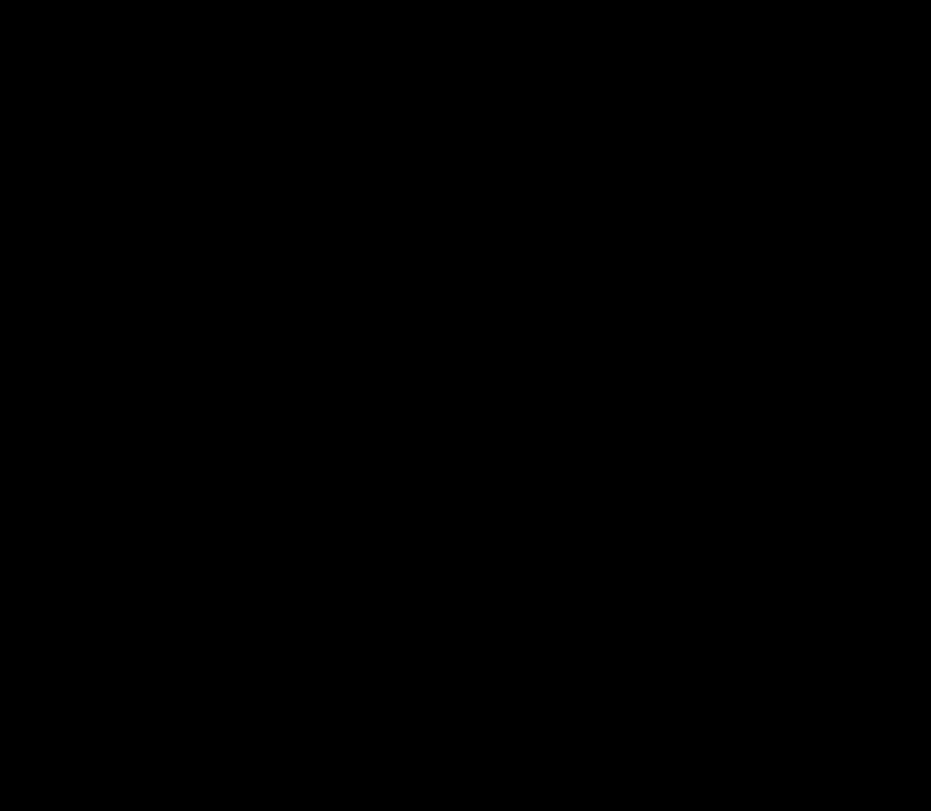

Supplement: Figure S6 — Comparison of genome copy numbers of mutant (MT) and wild-type (WT) virus after intra-thoracic infection of Ae. aegypti . Ten female mosquitoes were inoculated intra-thoracic with 0.17 µl of WT DENV-2 or E217A at a titer of 105 PFU/ml. Seven days later mosquitoes were killed by freezing and homogenized. Viral RNA was quantified by real-time qRT-PCR. Mean and 95% CI intervals are indicated by horizontal bars, each point represents a single female mosquito. P = 0.105, unpaired t test. (TIF) [file ppat.1003521.s006.tif]
