# Supplementary material for: Rational Design of a Live Attenuated Dengue Vaccine: 2′-O-Methyltransferase Mutants Are Highly Attenuated and Immunogenic in Mice and Macaques
Source: PLoS Pathog. 2013 Aug 1;9(8):e1003521. doi: 10.1371/journal.ppat.1003521 (PMC3731252; doi:10.1371/journal.ppat.1003521)
Supplement: Table S1 — SNPs of virus recovered from mice at day 3 after infection. (DOCX) [file ppat.1003521.s007.docx]

## Table S1

SNPs of virus recovered from mice at day 3 after infection.

DENV-1 SNPs

| Virus sample | position | Reference Base | Alternative Base | % coverage | Variant Quality | depth | p value (log10) |
| --- | --- | --- | --- | --- | --- | --- | --- |
| E216A in | 8220 | A | C | 99.82 | 189 | 5625 | -282 |
| E216A in | 8221 | A | C |  | 198 | 5651 | -282 |
| E216A out 1 | 8220 | A | C | 99.57 | 47.1 | 27 | -45 |
| E216A out 1 | 8221 | A | C |  | 36.3 | 27 | -42 |
| E216A out 2 | 8220 | A | C | 99.59 | 120 | 106 | -90 |
| E216A out 2 | 8221 | A | C |  | 127 | 106 | -93 |
| E217A+E216A out 1 | 8220 | A | C | 99.55 | 57.1 | 32 | -48 |
| E217A+E216A out 1 | 8221 | A | C |  | 66 | 32 | -51 |
| E217A+E216A out 2 | 8220 | A | C | 99.57 | 36.1 | 74 | -48 |
| E217A+E216A out 2 | 8221 | A | C |  | 45 | 75 | -54 |

DENV-2 SNPs

| Virus sample | position | Reference Base | Alternative Base | % coverage | Variant Quality | depth | p value (log10) |
| --- | --- | --- | --- | --- | --- | --- | --- |
| E217A in | 8219 | A | C | 99.77 | 199 | 5262 | -282 |
| E217A in | 8220 | G | C |  | 205 | 5195 | -282 |
| E217A out 1 | 8219 | A | C | 99.70 | 25.1 | 76 | -28 |
| E217A out 1 | 8220 | G | C |  | 60 | 74 | -51 |
| E217A out 2 | 8219 | A | C | 99.74 | 135 | 796 | -220 |
| E217A out 2 | 8220 | G | C |  | 143 | 788 | -277 |
| E217A+E216A out 1 | 8219 | A | C | 99.54 | 19 | 30 | -36 |
| E217A+E216A out 1 | 8220 | G | C |  | 13.2 | 28 | -36 |
| E217A+E216A out 2 | 8219 | A | C | 99.62 | 31.1 | 61 | -45 |
| E217A+E216A out 2 | 8220 | G | C |  | 35.1 | 60 | -48 |

In: virus input; out: virus output, position: position in genome; % coverage: % bases in the genome that were covered by at least one mapped read; Variant Quality: The Phred-scaled average quality score for the variant position; depth: number of reads mapped to the variant position; p-value: the negative Phred-scaled probability of the variant being homozygous.
